# Penile Metastases After Cystoprostatectomy for Urothelial Carcinoma of the Bladder: A Case Report

**DOI:** 10.7759/cureus.50767

**Published:** 2023-12-19

**Authors:** Touil Mohammed Amine, Mohamed Mokhtari, Elhadi Irsani, Youness Tahri, Hammou El Farhaoui, Anouar El Moudane, Ali Barki

**Affiliations:** 1 Urology, Mohammed VI University Hospital Center, Oujda, MAR; 2 Urology, Mohamed I University, Faculty of Medicine and Pharmacy, Oujda, MAR; 3 Urology, Centre Hospitalier Universitaire (CHU) Mohammed VI, Oujda, MAR; 4 Urology, Mohamed I University, Oujda, MAR

**Keywords:** metastasis, bladder cancer, corpora cavernosa, urothelial carcinoma, penile metastasis

## Abstract

Penile metastases from urothelial carcinoma are rare (1-8%). They most often (65%) occur within 18 months of diagnosis of the primary lesion and their prognosis is poor, with survival rarely exceeding 20 months. Treatment of cavernous metastases is multidisciplinary. The best results in terms of overall survival have been obtained with amputation of the penis combined with chemotherapy. We present a case of a 62-year-old male who presented with a metastasis of the penis. This was confirmed by MRI and biopsy, which confirmed the urothelial origin of the metastasis. The patient had undergone radical cystoprostatectomy for an invasive bladder tumor six months earlier. The patient died 10 days after the biopsy due to a significant deterioration in his general condition and the onset of consciousness disorders.

## Introduction

The occurrence of corpora cavernosa metastases is a rare event in the course of urothelial carcinoma of the blender, depending on the series, its incidence is estimated to be between 1% and 8% [1]. The diagnosis is confirmed by biopsy, which reveals the urothelial origin, cavernous metastases most often develop within 18 months of primary diagnosis (65%) and are usually associated with advanced disease [1]. The routes used by tumor cells to disseminate into the corpora cavernosa are still debated but are probably multiple, direct extension by contiguity, diffusion by arterial or venous route, or retrograde by lymphatic route [1]. A traumatic iatrogenic mechanism is discussed. In the literature, no predictive factor has been found to suggest a risk of developing cavernous metastases. Only Haddad mentions radiotherapy, which could favor the appearance of cavernous metastases in the course of bladder cancer [1]. The prognosis for these lesions is generally unfavorable, even when the treatment is poor, and with survival rarely exceeding 20 months [1]. We report a case of cavernous metastasis associated with multiple visceral metastases of urothelial carcinoma of the bladder after cystoprostatectomy diagnosed in a very advanced stage in a patient with general impairment.

## Case presentation

A 62-year-old male chronic smoker, with no medical or surgical history, followed in our institution for a urothelial carcinoma infiltrating the bladder muscle (pT2N0M0), preoperative thoracic, abdominal, and pelvic computed tomography (CT) showed no signs of suspicious nodal involvement nor distant metastatic spread. He has benefited from a cystoprostatectomy with bilateral ilio-obturator lymph node dissection and urinary diversion by ileal conduit type Bricker. The postoperative follow-up was simple without postoperative complications. Six months after the cystoprostatectomy, the patient experienced pain with discomfort at the base of the penis and a very important alteration of the general state. Clinical examination found a hard and fixed mass at the base of the penis at the level of the corpora cavernosa, without localized inflammatory signs.

**Figure 1:**
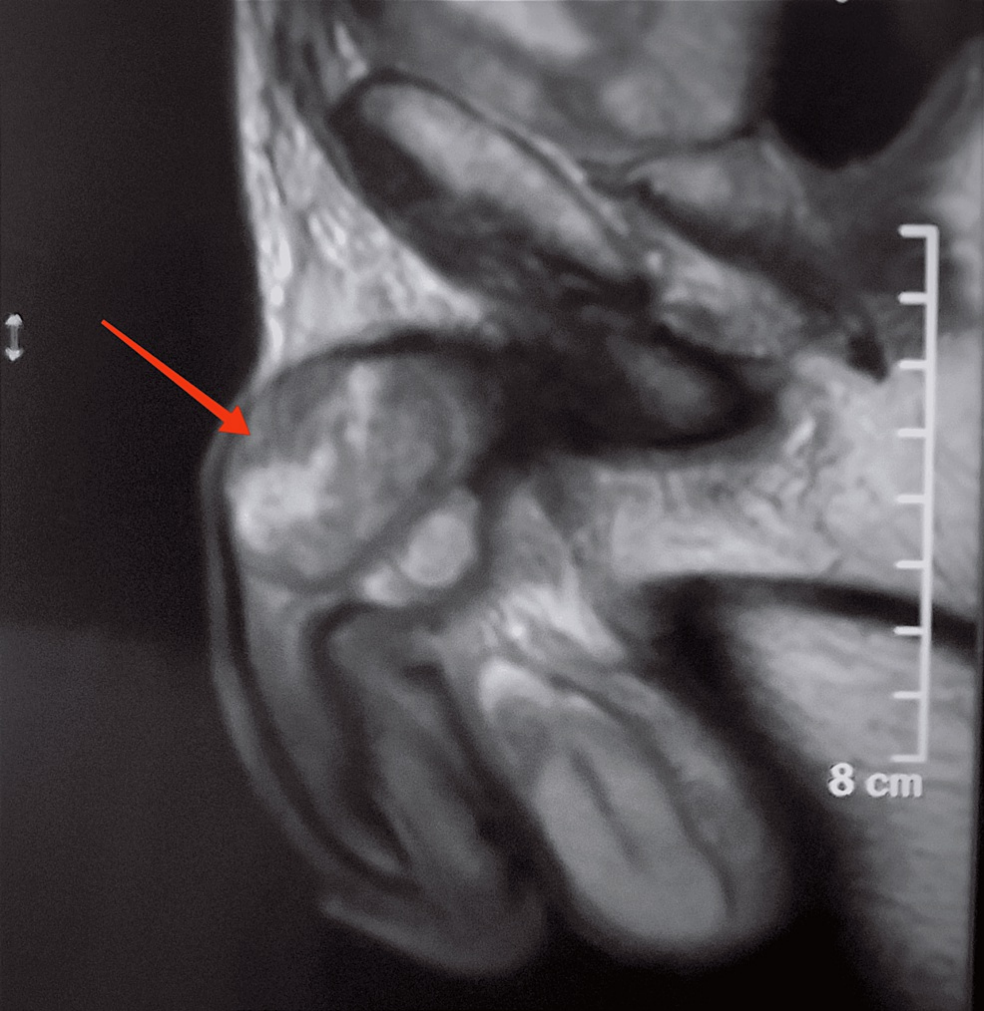
Pelvic MRI showing a nodular aspect infiltrating the corpora cavernosa without involvement of the corpus spongiosum or the urethra, with a tumoral appearance.

**Figure 2:**
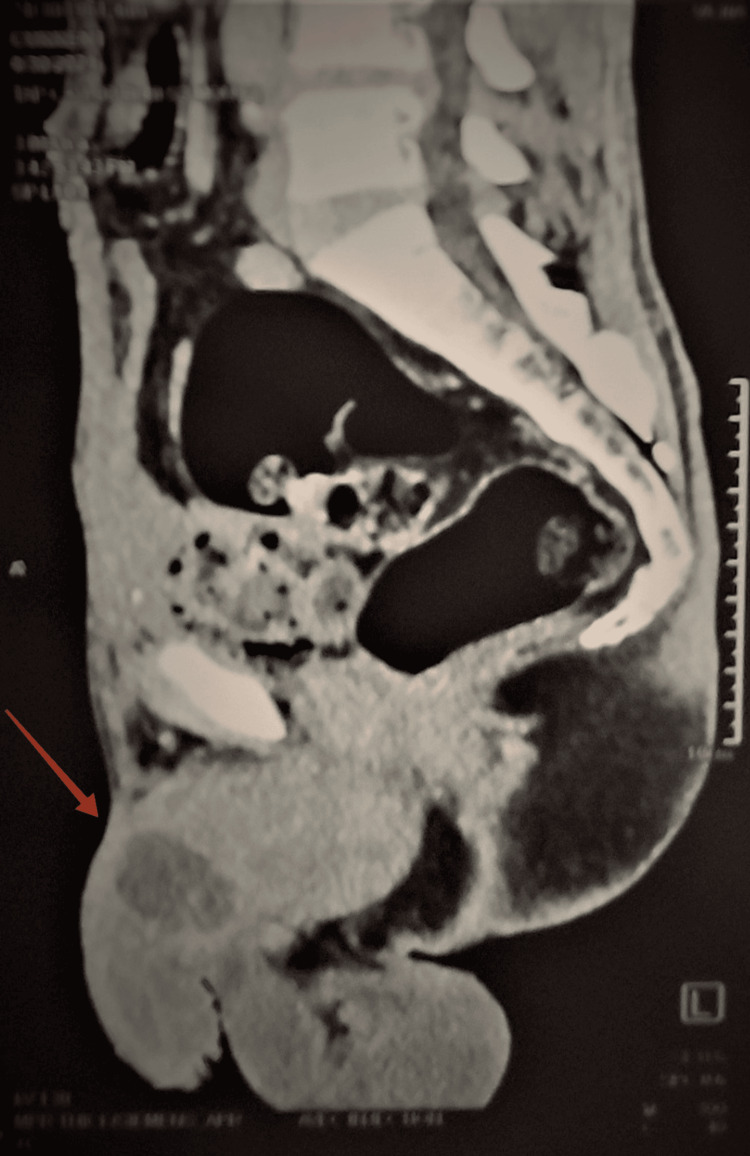
Radiographic aspect of the metastasis.

Pelvic MRI showed nodular infiltration of the corpora cavernosa without involvement of the corpus spongiosum or the urethra (Figure 1). A thoracic-abdominal-pelvic CT scan in search of other secondary locations revealed several liver and lung metastases in addition to the cavernous metastasis (Figure 2).

**Figure 3:**
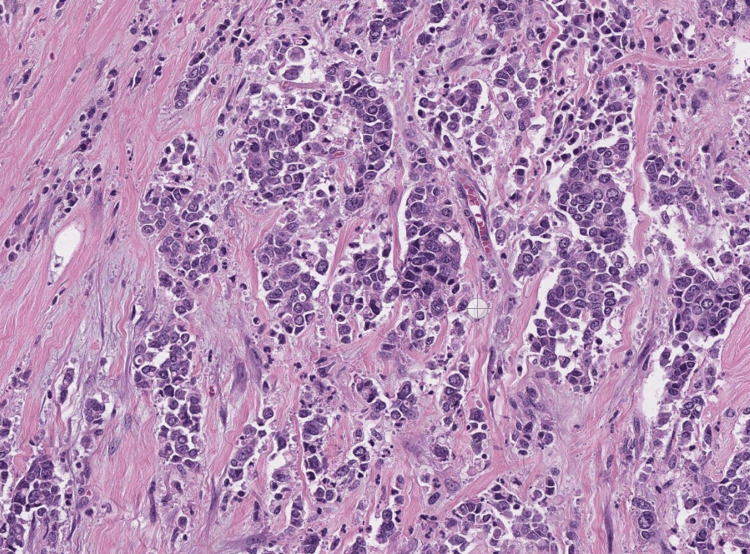
Microphotography showing the presence of carcinomatous nests and cords, surrounded by a desmoplastic stromal reaction. Few vascular clefts can also be seen.

A biopsy of the cavernous nodule was performed, revealing the existence of metastases of urothelial carcinoma on anatomopathological examination. The microphotography shows the presence of carcinomatous nests and cords surrounded by a desmoplastic stromal reaction. Few vascular clefts can be seen (Figure 3).

The patient died 10 days after the biopsy due to the significant deterioration of his general condition and the onset of consciousness.

## Discussion

Penile metastases from urothelial carcinoma of the bladder are rare (1-8%) [1]. They develop more in the corpus cavernosum than in the corpus spongiosum and most often occur within two years of the discovery of the primary cancer [2]. Penile localization usually presents with pain followed by cavernous nodular induration. Priapism was found in 38% of cases [1]. Other rarer signs such as acute urinary retention, dysuria, hematuria, or penile ulcers with superinfection can be found during metastasis of the corpora cavernosa, often delaying the diagnosis of these lesions [1]. Although penile metastases are easily identified on clinical examination, imaging is often necessary to assess the extent and degree of cavernous infiltration and to guide treatment.

Magnetic resonance imaging (MRI) is the gold standard imaging test for evaluating penile metastases [3]. It allows visualization of cavernous metastases, which present as inhomogeneous lesions with T1 hyposignal and T2 hypersignal. It also allows an accurate assessment of inguinal invasion and urethral obstruction that may be associated with metastatic nodules.

The discovery of cavernous metastases is rarely isolated and often associated with other metastases. The treatment of these metastases is difficult and multidisciplinary, symptomatic, and etiological. The etiological treatment depends on the primary cancer and its sensitivity to different chemotherapies. Other treatments are symptomatic, using radiotherapy alone or in combination with thermotherapy. Local infiltration of local anesthetics has a good effect on pain and on 30% of neoplastic priapism [4]. Surgical excision is indicated when the penile metastasis is single and limited [5]. Even with wide surgical excision and chemotherapy, the prognosis of penile metastases of urothelial carcinoma remains poor.

## Conclusions

In conclusion, the present case highlights the importance of close follow-up of patients who have undergone total cystoprostatectomy for muscle-invasive urothelial bladder cancer. Follow-up should include a complete physical examination of the whole body for atypical adenopathies skin lesions, priapism, the appearance of lower urinary tract symptoms, or haematuria. Follow-up must be tailored to each patient's general condition and the pathological stage of the disease. Recurrences may occur as early as a few months after the operation.

Cavernous metastases of urothelial carcinoma are rare and have a poor prognosis. They often occur at the same time as the general course of the disease. Treatment is multidisciplinary, but results are disappointing, with survival rarely exceeding two years. Curative surgery is only in the case of penile metastases single, limited penile metastases. The best results in terms of overall survival have been reported with total amputation of the penis and chemotherapy.
